# Utility of the Heat Index in defining the upper limits of thermal balance during light physical activity (PSU HEAT Project)

**DOI:** 10.1007/s00484-022-02316-z

**Published:** 2022-07-01

**Authors:** Daniel J. Vecellio, S. Tony Wolf, Rachel M. Cottle, W. Larry Kenney

**Affiliations:** 1 Center for Health Aging, College of Health and Human Development, Pennsylvania State University, 422 Biobehavioral Health Building, University Park, PA 16802 USA; 2 Department of Kinesiology, Pennsylvania State University, University Park, PA 16802 USA; 3 Graduate Program in Physiology, Pennsylvania State University, University Park, PA 16802 USA

**Keywords:** heat index;  wet bulb globe temperature;  universal thermal comfort index;  human thermoregulation;  critical environmental limits

## Abstract

Extreme heat events and consequent detrimental heat-health outcomes have been increasing in recent decades and are expected to continue with future climate warming. While many indices have been created to quantify the combined atmospheric contributions to heat, few have been validated to determine how index-defined heat conditions impact human health. However, this subset of indices is likely not valid for all situations and populations nor easily understood and interpreted by health officials and the public. In this study, we compare the ability of thresholds determined from the National Weather Service’s (NWS) Heat Index (HI), the Wet Bulb Globe Temperature (WBGT), and the Universal Thermal Climate Index (UTCI) to predict the compensability of human heat stress (upper limits of heat balance) measured as part of the Pennsylvania State University’s Heat Environmental Age Thresholds (PSU HEAT) project. While the WBGT performed the best of the three indices for both minimal activities of daily living (MinAct; 83 W**·**m^−2^) and light ambulation (LightAmb; 133 W**·**m^−2^) in a cohort of young, healthy subjects, HI was likewise accurate in predicting heat stress compensability in MinAct conditions. HI was significantly correlated with subjects’ perception of temperature and humidity as well as their body core temperature, linking perception of the ambient environment with physiological responses in MinAct conditions. Given the familiarity the public has with HI, it may be better utilized in the expansion of safeguard policies and the issuance of heat warnings during extreme heat events, especially when access to engineered cooling strategies is unavailable.

## Introduction

Extreme heat events have been on the rise for decades (Perkins et al. 2012; Perkins-Kirkpatrick and Lewis 2020) and are expected to continue to increase in frequency, duration, and intensity with continued climate change (Meehl and Tebaldi 2004; Russo et al. 2014; Vogel et al. 2020). They are associated with detrimental human health outcomes (Ebi et al. 2021) with links to spikes in morbidity (Sun et al. 2021; Wald 2019) and mortality (Anderson and Bell 2011; Fouillet et al. 2006; Whitman et al. 1997). Many environmental indices have been developed to quantify heat stress, not only through the determination of ambient dry-bulb temperature (*T*_db_) but also via additional effects of humidity, wind, and radiative gain (de Freitas and Grigorieva 2015). Many, but not all, of these indices were developed for, or have been used in the application of weather effects on human physiological responses.

The National Weather Service’s (NWS) Heat Index (HI), widely used in the USA, combines temperature and relative humidity to create a “feels-like” measure of the ambient environment (Rothfusz 1990). It is a simplified version of Steadman’s sultry index/apparent temperature (Steadman 1979a, 1979b, 1984), which took other simplified biometeorological and physiological parameters into account. However, the calculation of HI itself does not include information about air movement or radiant heat, limiting its usefulness in physiological applications. The HI began to be used operationally at the NWS in 1984 and is the current basis for their heat advisories and excessive heat warning products (Hawkins et al. 2017). Charts have been created, which denote caution, extreme caution, danger, and extreme danger zones that are associated with the risk of heat cramps, heat exhaustion, and heat stroke (NWS 2021), though it is unclear how the thresholds were determined. Although heat acclimatization is partially considered by differing thresholds for communities in the northern and southern USA, the HI and its thresholds are not based on physiological principles or empirical physiological data.

Other indices are more complete in their ability to describe physiological processes given that they incorporate additional environmental measures that play a role in human heat balance. The wet bulb globe temperature (WBGT) (Budd 2008) combines measurements from three thermometers (black globe, wet bulb, and dry bulb) to be able to account for not only air temperature and humidity as the HI does but also solar radiation’s impact on heat gain. It also indirectly accounts for the effects of wind. It was originally created to maintain heat safety amongst American military members during basic training drills (Yaglou and Minard 1956) but has become increasingly popular in other technical applications of heat safety. For example, in athletics, some American high schools have implemented WBGT-based thresholds for safe American football practice and play (Grundstein et al. 2015) while FIFA, soccer’s governing body, uses WBGT guidelines for determining required water breaks during matches (Mountjoy et al. 2012). The National Institute for Occupational Safety and Health (NIOSH) uses the WBGT to ensure safe working conditions in extreme heat (Jacklitsch et al. 2016). Another popular index known as the Universal Thermal Climate Index (UTCI) (Fiala et al. 2012; Jendritzky et al. 2012) has become increasingly employed over the past decade, especially in European studies, in the field of heat stress prediction given its complexity and completeness in describing the human heat budget. The UTCI utilizes a complete suite of atmospheric measures related to the environment’s impact on heat gain (air temperature, humidity, wind speed, and solar radiation) and incorporates them into a biophysical, thermoregulatory model (Fiala et al. 2012) with considerations made for metabolic workloads and clothing type to predict a range of conditions at which heat stress occurs (Bröde et al. 2012). Previous work has noted the suitability of the UTCI to be modeled properly for forecasting heat-health hazards (Di Napoli et al. 2018; Pappenberger et al. 2015) as well as its usefulness in defining heat waves given a heat-health focus (Di Napoli et al. 2019).

HI has been used in previous heat-health studies with varying success in predicting adverse outcomes. Weinberger et al. (2021) found HI-based heat alerts were associated with increases in hospitalizations for conditions such as heat stroke or fluid and electrolyte disorders but were not associated with increased all-cause mortality for those over 65 years old. This points to the effectiveness of the HI and HI-based warnings imparting behavioral responses when hazardous heat symptoms begin to develop. On the other hand, in a study investigating hyperthermia deaths in American football players, Grundstein et al. (2012) showed that mortality was associated with values of HI deemed a low threat for heat-related illness, while another index, the wet bulb globe temperature (WBGT), performed much better in predicting heat-related deaths, likely due to the WBGT’s ability to account for radiative heat gain. While other past studies have examined the ability to use HI in industrial safety settings (Bernard and Iheanacho 2015), to our knowledge there has been no study, which determines their suitability in predicting heat stress compensability (upper limits of human heat balance) with empirically gathered data in low-activity settings.

Here, we use empirical data from environmental chamber human subject studies from the ongoing PSU HEAT (Penn State University-Human Environmental Age Thresholds) project to determine the effectiveness of the HI in predicting the critical limits of heat stress—combinations of ambient temperature and humidity—in young, healthy adults. We compare environmental thresholds based on the HI, WBGT, and UTCI, keeping in mind differences in index input variables and calculation complexity. We also link physiological measures to subject perception as a way to help determine the underpinnings of how we may be able to combine environmental, physiological, and biobehavioral concepts to improve heat-health outcomes for all.

## Methods

### Subjects

Data were collected at Pennsylvania State University. All experiments were approved by the university’s ethics board and conformed to the guidelines set forth by the Declaration of Helsinki. Subjects were informed of experiment details during an initial screening process and, after explanation, gave oral and written informed consent for participation.

This study included 27 subjects, each completing between 2 and 6 experimental trials. Subjects were young, healthy men and women who were not taking any medications that would influence their thermoregulatory responses. They were normotensive, and no smokers were included in the study. There was no attempt made to control the menstrual cycle or contraceptive use. All participants wore standardized clothing during experiments, which included a lightweight cotton T-shirt, shorts, socks, and sneakers. Women participants also wore sports bras as part of their ensembles.

In a session prior to participating in experimental trials, all subjects underwent a maximal oxygen consumption (V̇O_2max_; mL kg^−1^ min^−−1^) test to quantify aerobic fitness. Testing was done using indirect calorimetry (Parvo Medics TrueOne® 2400, Parvo, UT, USA) during a maximal graded exercise test performed on a motor-driven treadmill (Bruce et al. 1973). Maximal V̇O_2_ was confirmed by (1) identifying a plateau in V̇O_2_ with increasing workloads, (2) a respiratory exchange ratio (RER) ≥ 1.10, and/or (3) a heart rate ≥ 90% of age-predicted maximal heart rate.

### Procedures

Subjects were given instructions to arrive well-hydrated and upon arriving for the experimental trials, subjects provided a urine sample to ensure adequate hydration, defined as urine specific gravity ≤ 1.020 (USG; PAL-S, Atago, Bellevue, WA) (Kenefick and Cheuvront 2012). No additional fluids were provided during the protocol. Subjects were asked to abstain from caffeine for at least 12 h and alcohol consumption for at least 24 h prior to the experiment. Additionally, subjects were asked to not participate in vigorous exercise for at least 24 h prior to testing.

During an individual visit, subjects participated in either a very light biking or walking trial, which represented the metabolic activity of the minimal activities of daily living (MinAct) or light ambulatory activity (LightAmb), respectively (Ainsworth et al. 2000). Experimental trials typically lasted between 60 and 120 min. MinAct trials involved pedaling on a cycle ergometer with zero resistance at a cadence of 40–50 rpm, while LightAmb trials consisted of walking on a motor-driven treadmill at 2.2 mph at a 3% grade. The individual trials were conducted in one of six transient environmental conditions comprising three critical water vapor pressure (*P*_crit_) and three critical dry-bulb temperature (*T*_crit_) trials. In *P*_crit_ trials, *T*_db_ was held constant at either 36, 38, or 40 °C, while in *T*_crit_ trials, *P*_a_ was held constant at 12, 16, or 20 mmHg. Chamber conditions were held constant for a 30-min equilibration period at the beginning of the experimental trial. After the equilibration period, *T*_db_ (for *P*_crit_ trials) or *P*_a_ (for *T*_crit_ trials) was raised by 1 °C or 1 mmHg every 5 min until a clear inflection and subsequent rise in the subject’s core temperature (*T*_c_) was observed. The ambient *T*_db_ and *P*_a_ at the time of *T*_c_ inflection were considered the critical environmental limits for heat stress compensability below, which subjects are in thermally safe conditions and above which their bodies no longer have the ability to physiologically maintain a stable core temperature. We have demonstrated excellent validity and reliability of this experimental protocol (Cottle et al. 2021). There was no solar radiation substitute or forced air movement inside the environmental chamber during experimental trials, though both do impact the human heat balance (Foster et al. 2022a, 2022b).

### Measurements

*T*_c_ was measured using gastrointestinal temperature telemetry capsules (VitalSense, Philips Respironics, Bend, OR, USA), which were taken by subjects 1–2 h before their experimental trial (Notley et al. 2021). *T*_c_ data were continuously measured and transmitted via Bluetooth to a PowerLab data acquisition system and LabChart signal processing software (AD Instruments, Colorado Springs, CO, USA) using an Equivital wireless physiological monitoring system (Equivital Inc., New York, NY, USA), which was placed in a harness that subjects wore on their upper torso.

During trials, V̇O_2_ and respiratory exchange ratio (RER; unitless) were measured 5 and 60 min after the onset of the trial. These were used to calculate the subject’s metabolic rate (*M*, Watts (W)), which was then normalized to body surface area:

$$M={\dot{\mathrm{V}}\mathrm{O}}_{2}\bullet \frac{\left[\left(\left(\frac{\mathrm{RER}-0.7}{0.3}\right)\bullet 21.13\right)+ \left(\left(\frac{1.0-\mathrm{RER}}{0.3}\right)\bullet 19.62\right)\right]}{60}\bullet 1000\bullet {{A}_{\mathrm{D}}}^{-1}$$

where *A*_D_ is the Dubois surface area in m^2^. In LightAmb (walking) trials, external work (*W*; W/m.^2^) was calculated as

$$W= 9.81\bullet m\bullet v \bullet {F}_{\mathrm{g}} \bullet {{A}_{\mathrm{D}}}^{-1}$$

where *m* is the subject’s body mass (kg), *v* is the walking velocity (m/min) and *F*_g_ is the grade of the treadmill expressed in fractional form. In the MinAct trials (biking), the external work *W* was assumed to be zero. Net metabolic heat production (*M*_net_) was the external work subtracted from the metabolic rate (Cramer and Jay 2019).

Subjects provided subjective perceptual data at 5, 30, 60, and 90 min as well as at the completion of their experimental trial. Subjects provided a rating of perceived exertion (RPE) based on the Borg 6–20 scale (Borg 1998). Additionally, subjects rated how hot (0–10 scale, 0 being neutral and 10 being unbearably hot) and humid (0–8 scale, 0 being neutral and 8 being unbearably humid) they felt the ambient environment was. As the total time in the chamber differed among subjects, this paper presents perceptual data at 5 and 30 min as well as at the completion of each trial.

### Heat stress indices

*T*_db_ and *T*_w_ were directly measured inside of the environmental chamber via mercurial thermometers encased in glass. These measures were then used to calculate the indices used in this study as described below.

The heat index (Rothfusz 1990) is calculated via a polynomial model with *T*_db_ (in °F) and relative humidity (0–100%) as inputs. It is computed as

$$Heat\;Index=-42.379+2.04901523T_{\mathrm{db}}+10.14333127\mathrm{RH}-0.22475541T_{\mathrm{db}}\ast\mathrm{RH}-6.83783\times10^{-3}\ast T_{\mathrm{db}}^2-5.481717\times10^{-3}\ast\mathrm{RH}^2+1.22874\times10^{-3}\ast T_{\mathrm{db}}^2\ast\mathrm{RH}+8.5282\times10^{-4}\ast T_{\mathrm{db}}\ast{RH}^2-1.99\times10^{-6}\ast T_{\mathrm{db}}^2\ast\mathrm{RH}^2$$

The WBGT is a combination of dry-bulb, wet-bulb, and globe temperature to assess heat stress due to atmospheric moisture content and radiation. No radiation lamps were used in the environmental chamber in this study, so the globe temperature was assumed to be equal to the dry-bulb temperature. Hence, indoor WBGT was calculated as

$$\mathrm{WBGT}=0.7{T}_{\mathrm{w}}+0.3{T}_{\mathrm{db}}$$

The UTCI was calculated using the pythermalcomfort package in Python 3.8. Inputs into the algorithm included dry-bulb temperature, mean radiant temperature, wind speed, and relative humidity. As above, the mean radiant temperature was assumed to be equal to the dry-bulb temperature due to the absence of any radiative source in the environmental chamber. There was no forced air movement in the chamber so wind speed was inputted as 0.5 m/s, the minimum wind speed valid for using the algorithm.

**Fig. 1:**
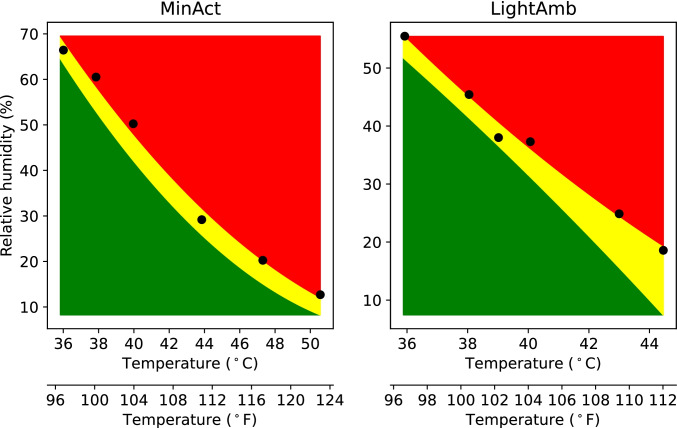
Compensability diagrams for MinAct and LightAmb scenarios based on empirical data collection from young, healthy subjects. Green regions in each diagram represent combinations of *T*_db_ and RH where heat stress is fully compensable. Red regions represent fully uncompensable scenarios. Yellow regions represent a transition or “danger” zone between the two modes of compensability. Dots represent the critical *T*_db_ and RH combinations for each of the six experimental conditions (updated from Wolf et al. (2021a))

To determine how well the thresholds of the three chosen indices dictate heat stress compensability based on the data collected as part of the PSU HEAT project, we plotted the categorical heat stress thresholds of the HI, WBGT, and UTCI against the critical environmental limits described above (Fig. 1, Table 1). The two highest thresholds of each index fell closest to the compensability limits dictated by our empirical data, so the analysis focused on these categorical values. Uncompensable heat stress was determined to be any *T*_db_–RH combination above the mean critical environmental limits from the study’s experimental trials (for more detail, see Wolf et al. (2021a)). To account for observational uncertainty and random errors, a second limit was created using the lower bound of the 95% confidence interval of the mean critical environmental limits at each environmental loci. T_db_–RH combinations below this second limit represent a zone of fully compensable heat stress where the body can successfully thermoregulate and maintain a stable core temperature for 97.5% of the young, healthy population. The area between these two limits represents a transitional “danger zone” where there is statistical uncertainty as to the compensability of heat stress.

**Table 1:** Upper two threshold categories (name and magnitude) for indices of interest: HI, WBGT, and UTCI

Index	Threshold category		
HI	Danger	103 °F	(NWS 2021)
	Extreme Danger	125 °F	
WBGT	Category 4	88 °F	(Yaglou and Minard 1956)
	Category 5	90 °F	
UTCI	Very strong heat stress	38 °C	(Błażejczyk et al. 2010)
	Extreme heat stress	46 °C	

## Results

### Subject characteristics

All subjects participated in both MinAct and LightAmb trials, though the number of times they performed in either trial differed. Subjects were young and healthy as shown by age, body surface area, and V̇O_2max_ (Table 2). As predicted a priori, oxygen consumption (V̇O_2_) and metabolic heat production (*M*_net_) were significantly lower in MinAct compared to LightAmb conditions (V̇O_2_ and *M*_net_: *p* < 0.001).

**Table 2:** Subject characteristics (mean ± standard deviation with the range in parentheses). Asterisks indicate significant differences between trials (*p* < 0.05)

Characteristic		
# of subjects	27 (13 M/14F)	
Age	24 ± 4 (18–34)	
Dubois body surface area (m^2^)	1.85 ± 0.2 (1.49–2.32)	
VO_2max_ (mL·kg^−1^·min^−1^)	49 ± 12 (29.7–79.1)	
	*MinAct*	*LightAmb*
VO_2_ (mL·kg^−1^·min^−1^)	0.46 ± 0.10* (0.30–0.69)	0.80 ± 0.16 (0.55–1.16)
*M*_net_ (W·m^−2^)	83.0 ± 12.5* (57.17–110.51)	133.3 ± 14.8 (102.77–173.53)

### Compensability

**Fig. 2:**
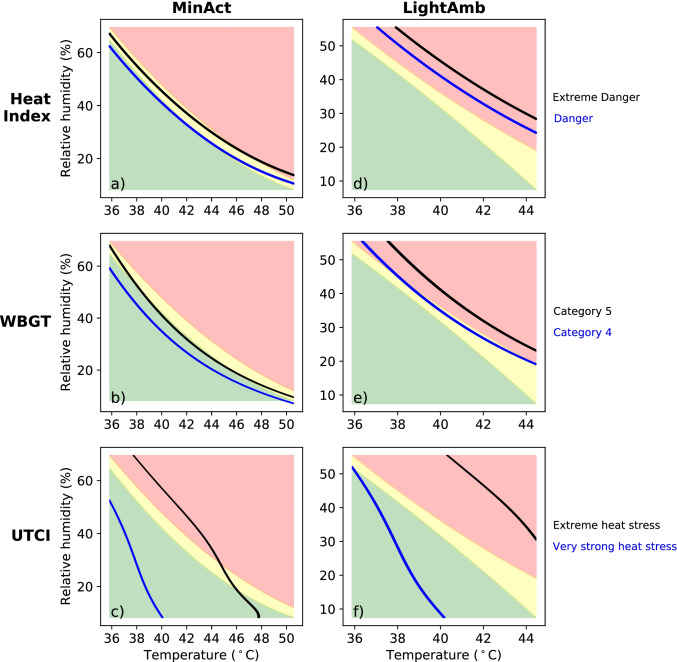
Compensability diagrams for the Heat Index, WBGT, and UTCI in MinAct and LightAmb trials. Blue and black lines in each diagram represent second-to-last and last categorial thresholds associated with each index

Via a visual inspection of the relation between the thermal indices and empirically derived compensability zones, the upper two categorical thresholds of both HI and WBGT were suitable for describing heat stress compensability (upper limit of heat balance) under MinAct conditions (Fig. 2a, 2c). The “danger” threshold for the HI was nearly identical to the cutoff between full heat stress compensability, and the “danger zone” as defined by the 95th percentile of critical environmental limits except for very hot and dry conditions. Similarly, the “extreme danger” HI threshold sits closely on the boundary between the “danger zone” and fully uncompensable heat stress. Both upper WBGT thresholds, “Category 4” and “Category 5” were within heat stress compensability limits as determined by physiological data. However, when increasing the metabolic workload to that of LightAmb, both HI thresholds were in the uncompensable range (Fig. 2b).

While threshold curves for HI and WBGT were nearly parallel to the physiological heat stress compensability zone thresholds, even when shifted away from zone boundaries (Fig. 2a–d), UTCI thresholds for “very strong heat stress” and “extreme heat stress” were nearly linear and spaced much further apart than the upper HI and WBGT thresholds (Fig. 2c, 2f). While the “very strong heat stress” threshold lies in the compensable zone for both MinAct and LightAmb work rates, the “extreme heat stress” threshold is associated with uncompensable heat stress in nearly all situations across the two work rates, except for some hot-dry conditions in MinAct.

### Perceptual data

**Fig. 3:**
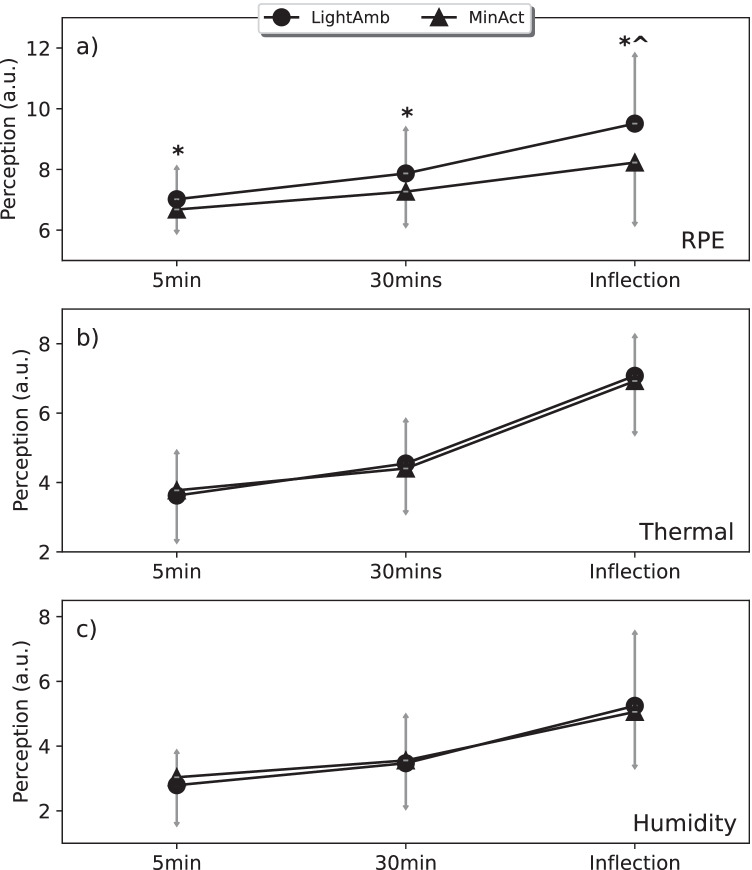
Average participant perceptual measure values. *Significant difference between MinAct and LightAmb at a time step. ^Significant difference between MinAct and LightAmb change in 30 min/inflection average values

To further investigate the rationale for the HI’s ability to accurately predict heat stress compensability during minimal activity, connections between perceptual data and other physiological parameters were studied. Statistical differences between MinAct and LightAmb RPEs were observed at 5 min (MinAct: 6.68, LightAmb: 7.02, *p* = 0.04), 30 min (MinAct: 7.27, LightAmb: 7.87, *p* = 0.01), and at core temperature inflection (MinAct: 8.23, LightAmb: 9.51, *p* = 0.002). Additionally, the increase in RPE between 30 min and core temperature inflection was statistically different between the two experimental work rates (MinAct: 0.98, LightAmb: 1.64, *p* = 0.03) (Fig. 3a). There were no statistical differences between MinAct and LightAmb at any of the three time points for thermal or humidity perception, nor in the change of thermal or humidity perception between the 30-min time point and core temperature inflection point (Fig. 3b, 3c).

Spearman correlations between the three perceptual measures and core and skin temperatures for both work rates are presented in Table 3. Skin temperatures were significantly correlated with thermal perception in both the MinAct (*r* = 0.34, *p* = 0.017) and LightAmb (*r* = 0.37, *p* = 0.007) trials. Additionally, in MinAct conditions, skin temperatures were significantly (negatively) correlated with humidity perception (*r* =  − 0.29, *p* = 0.044). During MinAct trials, participants again exhibited a significant relation between core temperature and thermal perception (*r* = 0.39, *p* = 0.009), while LightAmb participants’ core temperatures were significantly related to RPE (*r* = 0.32, *p* = 0.021).

**Table 3:** Spearman’s rank correlations between perceptual measures and skin/core temperatures at the time of core temperature inflection. Bolded values represent significance at the 95% confidence level

Thermal perception		Humidity perception		Perceived exertion	
Skin temperature					
*MinAct*	*LightAmb*	*MinAct*	*LightAmb*	*MinAct*	*LightAmb*
**0.34**	**0.37**	** − 0.29**	− 0.15	0.02	0.08
Core temperature					
*MinAct*	*LightAmb*	*MinAct*	*LightAmb*	*MinAct*	*LightAmb*
**0.37**	0.06	0.21	0.11	0.17	**0.32**

## Discussion

In this study, we set out to determine if the widely promulgated HI was useful in determining heat stress compensability as defined by empirically defined thresholds for uncompensable heat stress. In MinAct settings, the “danger” and “extreme danger” thresholds established by the NWS (2021) closely follow the upper limit of compensability as defined by the empirically derived *T*_db_–RH relation. This was not true for the increased activity associated with LightAmb conditions. It is well-documented that increased metabolic activity lowers critical environmental limits when quantified by temperature or another index such as WBGT (Vecellio et al. 2021; Wolf et al. 2021a, 2021b). We conclude that the HI adequately describes physiologically determined upper limits of heat balance during minimal metabolic workloads, but that the addition of anything more than this limited internal heat production causes deviations from efficacious HI utility in this regard. Additionally, we reiterate the lack of the effect of solar radiation on our results. Not only is this a significant part of the heat gain equation, but the HI also does not account for its effects in its calculation. Hence, the results of this study are most useful and applicable for use indoors or in the shade in outdoor situations as has been noted in previous work (Foster et al. 2021).

While not the focus of this study, we also note that these compensability curves would likely shift in the event they were developed given a different population of subjects. For example, the compensability limits associated with elderly participants would likely be shifted downward from where they stand in Fig. 1 due to impairments in the physiological mechanisms dictating the maintenance of thermal balance (Kenney and Munce 2003). Heat acclimatization, or acclimation, has been shown to improve sweating efficiency, which may provide a positive shift in compensability curves, likely in the hot-dry region where evaporative cooling is a limiting factor (Baker 2019; Klous et al. 2020). External factors also play a role. All subjects in this study wore a standard outfit of T-shirts, shorts, and shoes, but increased or decreased clothing coverage or material changes would affect thermal and moisture diffusivity and subsequent heat transfer (Havenith 2002). However, the cohort used in this study (young, healthy subjects) provides a relative maximum tolerance to extreme heat for the average person.

The connections between perception and physiology in these results provide one perspective for the HI’s divergent activity-based predictive value. In the MinAct tests, both skin temperature and core temperature were correlated with thermal perception. Skin temperatures were also significantly correlated with humidity perception, likely due to the lack of forced convection creating an environment unconducive to the effective evaporation of sweat from the skin surface. These results indicate that a linkage between the perception of the ambient environment and the rise in core temperature exists in minimal activity settings. While skin temperature and thermal perception were also correlated in the LightAmb trials, only RPE correlated with core temperature. While study participants felt hot because they were in an extremely hot environmental chamber, the added metabolic heat production was the determining factor in the relation to core temperature. The relationship between perception, core temperature, and the metabolic workload was also borne out in examining the differences in perceptual measures between MinAct and LightAmb trials, in which only RPE was different (Fig. 3).

Apart from simple air temperature, the HI is likely the most well-known measure among the public for the impact or feel of the environment in terms of heat in the USA, given its prevalence and notoriety in warm-season weather forecasts from both public and private entities. As with nearly all heat measures in the face of climate change, research shows that extreme HI days are projected to increase across the USA by the end of the twenty-first century (Dahl et al. 2019). While it is used in the issuance of excessive heat warnings by the National Weather Service, these warnings are not based on human physiology. Instead, they set discrete values (generally 105 °F in the “north” and 110 °F in the “south” with some exceptions) as the thresholds for warnings to be activated (Hawkins et al. 2017), which differ from the health-based thresholds they promote on their other webpages (NWS 2021). We do not believe that our results are in dispute with the current excessive heat warning criteria, though. The excessive heat advisories and warnings put out by the National Weather Service are typically meant for those partaking in higher activity settings (i.e., outdoor work, recreation, or entertainment), which require a much lower threshold for improving heat-health outcomes. Rather, the results of this study may provide a second layer to warning communication and be based upon physiological thresholds of heat stress given (1) reduced work rates and (2) the worst-case scenario of prolonged heat exposure without access to cooling. For example, events such as the 2021 Pacific Northwest heatwave (Silberner 2021) caused many residents in the region to lose access to cooling mechanisms (air conditioning or electric fans) due to power outages. This caused many to perish or become intensely ill in their homes, all while likely being relatively inactive. Second-level warnings and associated policies could focus on evacuating residents in areas susceptible to power outages or that are typically without air conditioners to nearby cooling centers for safety until the heat breaks (Berisha et al. 2017; Widerynski et al. 2017). The effect of age should also be considered if these policy updates are made given the detrimental effects of aging on thermoregulation and the prevalence of elderly persons dying alone in their homes during these events.

While the WBGT performs just as well if not better than the HI in predicting heat stress compensability, it has primarily been used in determining thermal safety in athletic competitions (Grundstein et al. 2015) and industrial settings (Jacklitsch et al. 2016). Unlike the HI, the general public has little familiarity with WBGT and its uses. Additionally, WBGT adds a layer of complexity that limits its intuitive interpretation. For example, WBGT values are lower than the air temperature on extreme heat days, which may not make sense to end users of forecasts in decision-making processes. This is in contrast with the HI, which is typically higher than the air temperature, signifying to the lay public that the humidity makes it feel warmer than it is. Therefore, the use of HI for MinAct situations may be a useful tool in creating additional heat-health policies. Concurrently, given its predictive value in both MinAct and LightAmb conditions, science communicators should investigate ways of making the public more aware and how to understand the WBGT.

Results indicating that the UTCI heat stress thresholds were not suitable for predicting our empirically derived compensability limits were surprising, though potentially explainable. We highlight two possible reasons for the possible discrepancy:

**Heat stress basis:** The (Fiala et al. 2012) human thermoregulatory model is thorough in design and has been validated against other physiologically based indices over a range of environmental and work rate conditions (Psikuta et al. 2012). Dynamics of heat stress response were shown to be explained not only by core (rectal) temperature but also by skin temperature, sweat rate, skin wettedness, and skin blood flow amongst other physiological variables. We have previously shown that there was large variability in sweat rates and skin temperatures amongst subjects who completed these studies (Vecellio et al. 2021), which would likely differ from one calculated measure that would come from the Fiala model. Additionally, measures of all these variables at 30 and 120 min seemed to play an important role. Our analysis relied on core temperature inversion alone as the signifier of uncompensable heat stress. In Błażejczyk et al. (2013)’s categorization of UTCI heat stress, “extreme heat stress” is described by “an increase in the *T*_re_ [rectal temperature] gradient,” which is consistent with our definition but also includes quantified time-dependent sweat rates and net heat losses. “Very strong heat stress” responses are based upon 30-min measures of core temperature and skin temperature, well before any of the subjects from this study reached core temperature inflection.

**Work rates:** Fiala et al. (2012)’s thermoregulatory model was validated at a reference metabolic rate of 133 W m^−2^, similar to the workload sustained in this study’s LightAmb condition. A compendium of studies recently conducted by Foster et al. (2022a, 2022b) showed the advantage that the UTCI had over other indices due to its direct inclusion of thermal radiation and wind movement in predicting heat stress, but these studies were geared toward work capacities nearing 30% VO_2max_, well above the conditions of both of this study’s workload scenarios. This was also found in recent work by Ioannou et al. (2022). It is unclear if the UTCI has ever been tested in minimal activity scenarios such as the one presented here.

Given the considerations of how heat stress is quantified as described above and noting that our experiments did not include radiative and air movement influences, which the UTCI has been fine-tuned for, we believe that these factors may be the reason for the UTCI’s surprising lack of skill in predicting heat stress in this study. We do not see this as a deficiency in the metric but rather an avenue for future work in ensuring its effectiveness for minimal metabolic outputs.

There are also pathways for future research on the physiological impacts of the results presented here. All subjects included in this study had core temperatures well below the clinical threshold for heat stroke of 40 °C (Bouchama and Knochel 2002), meaning that reaching these limits is a starting point for the risk of severe heat-health ailments. Seminal work from Lind (1963) indicated that after body core temperature inflection, humans could remain in the heat for up to 8 h before falling out of the “prescriptive zone” while performing work. Future work should examine rates of heat storage after inflection is achieved to further our understanding of how long humans could safely stay in the heat if forced to, especially in a world of increased, sustained heat (Han et al. 2022) and the risk of infrastructure failure, which inhibits cooling mechanisms such as fans and air conditioning (Perera et al. 2020).

## Conclusion

Using critical environmental limits from well-controlled human subject experiments, the present paper determined that categorical thresholds associated with the widely used HI can be used to predict heat stress compensability under minimal activity conditions resembling the activities of daily living (fidgeting, self-care, showering, eating, dressing, washing dishes, etc.) in the absence of solar radiation. The utility of the Heat Index for determining thermally “safe” conditions is diminished as activity level, and therefore metabolic heat production, increases. While the wet bulb globe temperature is broadly used in determining safe conditions for industry and sport, results from this study present an alternative measure more intuitive to, and better understood by, the public. In addition to heat warning thresholds set by the National Weather Service, their current heat index categorical thresholds can be updated with this empirical, physiological-based information and communicated with the general population to improve heat-health outcomes during extreme heat events.

## References

[CR1] Ainsworth BE et al (2000) Compendium of physical activities: an update of activity codes and MET intensities. Med Sci Sports Exerc 32(9; SUPP/1):S498–S504 10.1097/00005768-200009001-00009 10993420

[CR2] Anderson GB, Bell ML (2011). Heat waves in the United States: mortality risk during heat waves and effect modification by heat wave characteristics in 43 US. Commu Environ Health Perspect.

[CR3] Baker LB (2019). Physiology of Sweat Gland Function: the Roles of Sweating and Sweat. Composition in Human Health Temperature.

[CR4] Berisha V (2017). Assessing adaptation strategies for extreme heat: a public health evaluation of cooling centers in Maricopa County. Arizona Weather, Climate, and Society.

[CR5] Bernard TE, Iheanacho I (2015). Heat index and adjusted temperature as surrogates for wet bulb globe temperature to screen for occupational heat stress. J Occup Environ Hyg.

[CR6] Błażejczyk K (2010). Principles of the new Universal Thermal Climate Index UTCI and its application to bioclimatic research in European scale Miscellanea. Geographica.

[CR7] Błażejczyk K (2013). An introduction to the Universal Thermal Climate Index UTCI. Geographia Polonica.

[CR8] Borg G (1998) Borg’s perceived exertion and pain scales. Borg's perceived exertion and pain scales. Human Kinetics, Champaign, IL, US. https://psycnet.apa.org/record/1998-07179-000

[CR9] Bouchama A, Knochel JP (2002). Heat Stroke New Engl J Med.

[CR10] Bröde P (2012). Deriving the operational procedure for the Universal Thermal Climate Index UTCI. Int J Biometeorol.

[CR11] Bruce RA, Kusumi F, Hosmer D (1973). Maximal oxygen intake and nomographic assessment of functional aerobic impairment in cardiovascular disease. Am Heart J.

[CR12] Budd GM (2008) Wet-bulb globe temperature (WBGT)—its history and its limitations J Sci Med Sport 11:20-32. 10.1016/j.jsams.2007.07.003 10.1016/j.jsams.2007.07.003 17765661

[CR13] Cottle RM, Wolf ST, Lichter ZS, Kenney WL (2021). Validity and reliability of a protocol to establish human critical environmental limits (PSU HEAT). J Appl Physiol.

[CR14] Cramer MN, Jay O (2019). Partitional Calorimetry. J Appl Physiol.

[CR15] Dahl K, Licker R, Abatzoglou JT, Declet-Barreto J (2019). Increased frequency of and population exposure to extreme heat index days in the United States during the 21st century. Environ Res Commun.

[CR16] de Freitas CR, Grigorieva EA (2015). A comprehensive catalogue and classification of human thermal climate indices. Int J Biometeorol.

[CR17] Di Napoli C, Pappenberger F, Cloke HL (2018). Assessing heat-related health risk in Europe via the Universal Thermal Climate Index UTCI. Int J Biometeorol.

[CR18] Di Napoli C, Pappenberger F, Cloke HL (2019). Verification of heat stress thresholds for a health-based heat-wave definition. J Appl Meteorol Climatol.

[CR19] Ebi KL (2021). Hot weather and heat extremes: health risks. The Lancet.

[CR20] Fiala D, Havenith G, Bröde P, Kampmann B, Jendritzky G (2012). UTCI-Fiala multi-node model of human heat transfer and temperature regulation. Int J Biometeorol.

[CR21] Foster J, Smallcombe JW, Hodder S, Jay O, Flouris AD, Havenith G (2022). Quantifying the impact of heat on human physical work capacity; part II: the observed interaction of air velocity with temperature, humidity, sweat rate, and clothing is not captured by most heat stress indices. Int J Biometeorol.

[CR22] Foster J, Smallcombe JW, Hodder S, Jay O, Flouris AD, Nybo L, Havenith G (2021). An advanced empirical model for quantifying the impact of heat and climate change on human physical work capacity. Int J Biometeorol.

[CR23] Foster J, Smallcombe JW, Hodder S, Jay O, Flouris AD, Nybo L, Havenith G (2022). Quantifying the impact of heat on human physical work capacity part III: the impact of solar radiation varies with air temperature humidity and clothing coverage. Int J Biometeorol.

[CR24] Fouillet A (2006). Excess mortality related to the August 2003 heat wave in France. Int Arch Occup Environ Health.

[CR25] Grundstein A, Williams C, Phan M, Cooper E (2015). Regional heat safety thresholds for athletics in the contiguous United States. Appl Geogr.

[CR26] Grundstein AJ (2012). A retrospective analysis of American football hyperthermia deaths in the United States. Int J Biometeorol.

[CR27] Han Q, Sun S, Liu Z, Xu W, Shi P (2022). Accelerated Exacerbation of Global Extreme Heatwaves under Warming Scenarios. Int J Climatol n/a.

[CR28] Havenith G (2002). Interact Cloth Thermo Exogen Dermatol.

[CR29] Hawkins MD, Brown V, Ferrell J (2017). Assessment of NOAA National Weather Service methods to warn for extreme heat events Weather. Clim Soc.

[CR30] Ioannou Ioannou LG et al. (2022) Indicators to assess physiological heat strain – Part 3: multi-country field evaluation and consensus recommendations. Temperature:1–18. 10.1080/23328940.2022.2044739 10.1080/23328940.2022.2044739 PMC9559325 36249710

[CR31] Jacklitsch B, Williams J, Musolin K, Coca A, Kim JH, Turner N (2016) Occupational exposure to heat and hot environments. US Department of Health and Human Services, Centers for Disease Control and Prevention, NIOSH, Cincinnati, OH, USA, pp 1–159

[CR32] Jendritzky G, de Dear R, Havenith G (2012). UTCI—Why another thermal index?. Int J Biometeorol.

[CR33] Kenefick RW, Cheuvront SN (2012). Hydration for recreational sport and physical activity. Nutr Rev.

[CR34] Kenney WL, Munce TA (2003). Invited review: aging and human temperature regulation. J Appl Physiol.

[CR35] Klous L, De Ruiter C, Alkemade P, Daanen H, Gerrett N (2020). Sweat rate and sweat composition during heat acclimation. J Therm Biol.

[CR36] Lind AR (1963). Physiological effects of continuous or intermittent work in the heat. J Appl Physiol.

[CR37] Meehl GA, Tebaldi C (2004) More intense, more frequent, and longer lasting heat waves in the 21st century Science 305:994–997. 10.1126/science.1098704 10.1126/science.1098704 15310900

[CR38] Mountjoy M, Alonso J-M, Bergeron MF, Dvorak J, Miller S, Migliorini S, Singh DG (2012) Hyperthermic-related challenges in aquatics, athletics, football, tennis and triathlon Br J Sports Med 46:80. 10.1136/bjsports-2012-091272 10.1136/bjsports-2012-091272 22906783

[CR39] Notley SR, Meade RD, Kenny GP (2021). Time following Ingestion Does Not Influence the Validity of Telemetry Pill Measurements of Core Temperature during Exercise-Heat Stress: the. Journal Temperature Toolbox Temperature.

[CR40] NWS (2021) What is the heat index? https://www.weather.gov/ama/heatindex. Accessed 22 October 2021

[CR41] Pappenberger F, Jendritzky G, Staiger H, Dutra E, Di Giuseppe F, Richardson DS, Cloke HL (2015). Global forecasting of thermal health hazards: the skill of probabilistic predictions of the Universal Thermal Climate Index UTCI. Int J Biometeorol.

[CR42] Perera ATD, Nik VM, Chen D, Scartezzini J-L, Hong T (2020). Quantifying the impacts of climate change and extreme climate events on energy systems Nature. Energy.

[CR43] Perkins SE, Alexander LV, Nairn JR (2012) Increasing frequency, intensity and duration of observed global heatwaves and warm spells Geophysical Research Letters 39. 10.1029/2012GL053361

[CR44] Perkins-Kirkpatrick SE, Lewis SC (2020). Increasing Trends in Regional. Heatwaves Nature Communications.

[CR45] Psikuta A (2012). Validation of the Fiala multi-node thermophysiological model for UTCI application. Int J Biometeorol.

[CR46] Rothfusz LP (1990) The heat index equation (or, more than you ever wanted to know about heat index). National Oceanic and Atmospheric Administration, National Weather Service, Office of Meteorology, Fort Worth, Texas, p 9023

[CR47] Russo S et al. (2014) Magnitude of extreme heat waves in present climate and their projection in a warming world J Geophys Res: Atmos 119:12,500–512. 10.1002/2014JD022098

[CR48] Silberner J (2021) Heat wave causes hundreds of deaths and hospitalisations in Pacific north west BMJ : Br Med J (Online) 374. 10.1136/bmj.n1696 10.1136/bmj.n1696 34226179

[CR49] Steadman RG (1979). The assessment of sultriness Part i: a Temperature-Humidity Index Based on Human Physiology and Clothing Science. J Appl Meteorol Climatol.

[CR50] Steadman RG (1979). The assessment of sultriness Part II: Effects of Wind, Extra Radiation and Barometric Pressure on Apparent Temperature. J Appl Meteorol Climatol.

[CR51] Steadman RG (1984). A universal scale of apparent temperature. J Appl Meteorol Climatol.

[CR52] Sun S, Weinberger KR, Nori-Sarma A, Spangler KR, Sun Y, Dominici F, Wellenius GA (2021). Ambient heat and risks of emergency department visits among adults in the United States: time stratified case crossover study. BMJ.

[CR53] Vecellio DJ, Wolf ST, Cottle RM, Kenney WL (2021) Evaluating the 35°C wet-bulb temperature adaptability threshold for young, healthy adults (PSU HEAT) J Appl Physiol 0:null 10.1152/japplphysiol.00738.2021 10.1152/japplphysiol.00738.2021 PMC8799385 34913738

[CR54] Vogel MM, Zscheischler J, Fischer EM, Seneviratne SI (2020) Development of future heatwaves for different hazard thresholds J Geophys Res: Atmos 125:e2019JD032070: 10.1029/2019JD032070 10.1029/2019JD032070 PMC7380308 32728502

[CR55] Wald A (2019). Emergency department visits and costs for heat-related illness due to extreme heat or heat waves in the United States: an integrated review. Nurs Econ.

[CR56] Weinberger KR et al. (2021) Heat warnings, mortality, and hospital admissions among older adults in the United States Environment International 157:106834. 10.1016/j.envint.2021.106834 10.1016/j.envint.2021.106834 PMC11549953 34461376

[CR57] Whitman S, Good G, Donoghue ER, Benbow N, Shou W, Mou S (1997) Mortality in Chicago attributed to the July 1995 heat wave Am J Public Health 87:1515–1518. 10.2105/ajph.87.9.1515 10.2105/ajph.87.9.1515 PMC1380980 9314806

[CR58] Widerynski S et al (2017) Use of cooling centers to prevent heat­related illness: summary of evidence and strategies for implementation. Centers for Disease Control and Prevention, Atlanta, GA

[CR59] Wolf ST, Cottle RM, Vecellio DJ, Kenney WL (2021a) Critical environmental limits for young, healthy adults (PSU HEAT) J Appl Physiol 0:null. 10.1152/japplphysiol.00737.2021a 10.1152/japplphysiol.00737.2021 PMC8799386 34913739

[CR60] Wolf ST, Folkerts MA, Cottle RM, Daanen HAM, Kenney WL (2021). Metabolism- and sex-dependent critical WBGT limits at rest and during exercise in the heat American Journal of Physiology-Regulatory. Int Com Physio.

[CR61] Yaglou CP, Minard D (1956) Prevention of heat casualties at Marine Corps training centers. Harvard School of Public Health, Boston. The document is available online at http://www.dtic.mil/dtic/tr/fulltext/u2/099920.pdf

