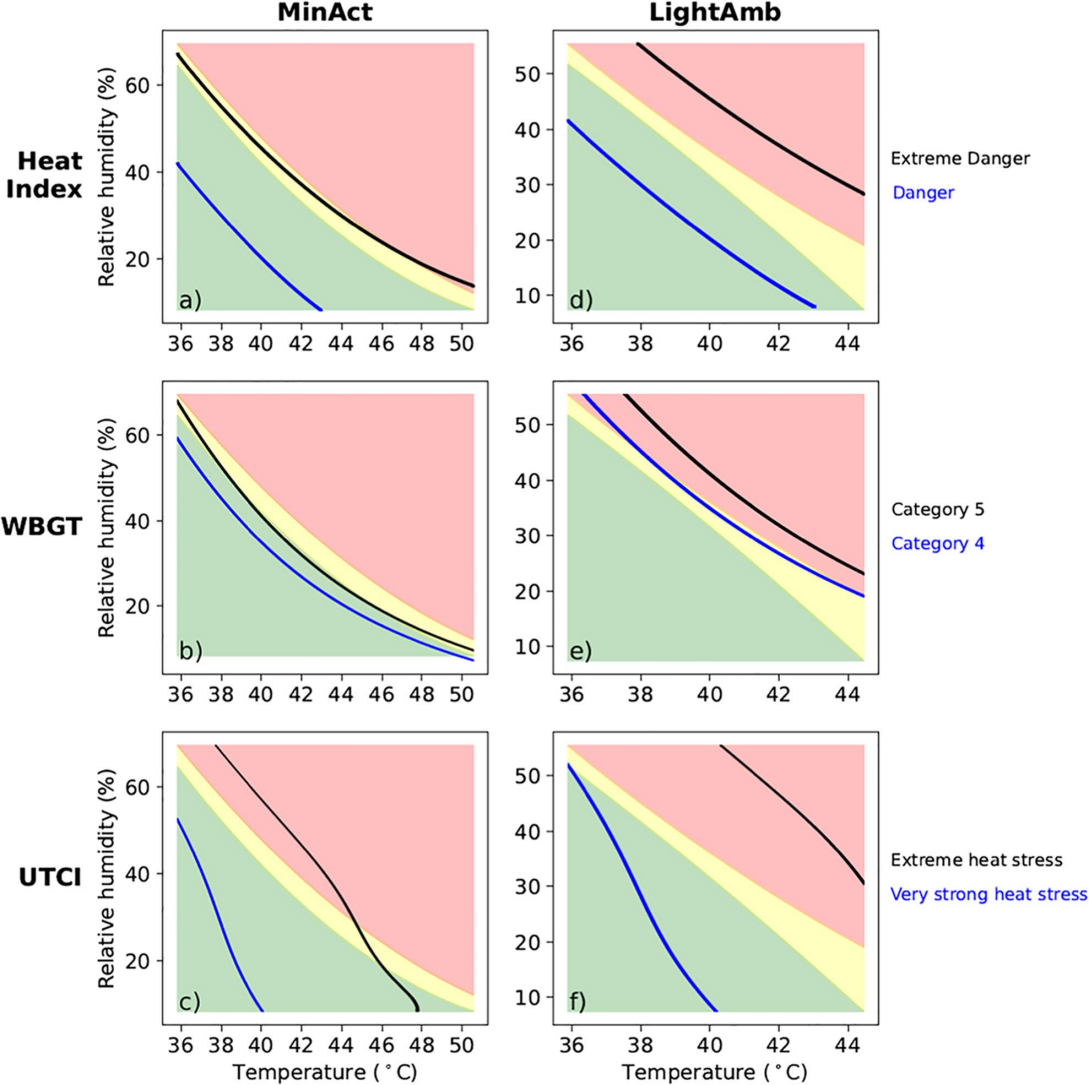# Correction to: Utility of the Heat Index in defining the upper limits of thermal balance during light physical activity (PSU HEAT Project)

**DOI:** 10.1007/s00484-022-02384-1

**Published:** 2022-10-21

**Authors:** Daniel J. Vecellio, S. Tony Wolf, Rachel M. Cottle, W. Larry Kenney

**Affiliations:** 1 Center for Health Aging, College of Health and Human Development, Pennsylvania State University, 422 Biobehavioral Health Building, University Park, PA 16802 USA; 2 Department of Kinesiology, Pennsylvania State University, University Park, PA 16802 USA; 3 Graduate Program in Physiology, Pennsylvania State University, University Park, PA 16802 USA

**Correction to: International Journal of Biometeorology (2022) 66:1759–1769**

**https://doi.org/10.1007/s00484-022-02316-z**

This corrections stands to correct the original article, where on page 1764, the lines representing “Danger” for the Heat Index in Fig. 2 a and d were misplaced. They are placed at a Heat Index of 120°F, which was used in a previous iteration of the code used for analysis, instead of the Danger threshold of 103°F. The correct Fig. 2 should be presented as below. Additionally, the description of the Danger threshold in the “Compensability” section of the results (lines 6–10 of the section) should be disregarded. This, however, does not change the main conclusions of the paper, as the “Extreme Danger” category of the Heat Index as defined by the National Weather Service is supported by empirical, physiological data in MinAct conditions.

The authors regret and apologize for the error.